# Comparing Cancer Primary and Secondary Prevention Documentation Between Different Digital Health Approaches in the Emergency Department

**DOI:** 10.1089/whr.2024.0104

**Published:** 2024-09-26

**Authors:** Sally K. Stauder, Shalmali R. Borkar, Anna Najor, Adrienne Hunter, Christopher DeStephano, Michael Mohseni

**Affiliations:** ^1^ Florida State University College of Medicine, Tallahassee, Florida, USA.; ^2^ Robert D. and Patricia E. Kern Center for the Science of Health Care Delivery, Mayo Clinic, Jacksonville, Florida, USA.; ^3^ Department of Obstetrics and Gynecology, Montefiore Medical Center, Bronx, New York, USA.; ^4^ Department of Medical and Surgical Gynecology, Mayo Clinic, Jacksonville, Florida, USA.; ^5^ Department of Emergency Medicine, Mayo Clinic, Jacksonville, Florida, USA.

**Keywords:** obstetrics/gynecology, electronic health records and systems, emergency medicine, cancer, preventive medicine, clinical information systems

## Abstract

**Background:**

Decreasing primary care access and increasing emergency department (ED) usage is a potential contributor to declining cancer screening prevalences in those facing barriers to health care access. The ED is a non-traditional yet potentially high-yield setting for implementation of interventions to monitor and increase cancer screening.

**Methods:**

An ED-administered survey in July 2022 gathered data on breast, cervical, and colorectal cancer screening, as well as human papillomavirus (HPV) vaccination status of females presenting to the ED for care. This was compared with electronic health record (EHR) data extraction of all ED patients during the same timeframe. Primary outcome was proportion of cancer screening and HPV vaccination not up to date in each group.

**Results:**

ED survey was administered to 101 individuals; EHR data was extracted on 2934 patients. Survey versus EHR, respectively, found cervical cancer screening was not up to date in 6.2% vs. 77.6%, breast cancer screening in 14.3% vs. 73.4%, colorectal cancer screening in 22.9% vs. 56.5%, and HPV vaccination in 33.3% vs. 57.8%. *p* value was < 0.001 for all screening category comparisons between survey and EHR.

**Discussion:**

Our data indicate significant discrepancies between self-reported screening history and EHR data. ED survey results were more in line with the observed screening rates in various surveillance systems and published in the literature. This suggests that point-of-care ED survey administration may be more effective in identifying those needing preventative cancer screening, especially in individuals with less access to routine health care.

## Introduction 

Mortality rates nationally are decreasing for cancers with guideline-recommended screening tests.^1,2^ These include breast, cervical, colon, and lung cancers.^3–6^ However, the benefits of cancer screening have been unevenly distributed in the population and efforts are underway to address equity and access to participation in cancer screening programs.^7^ One example is cervical cancer which is highly preventable in women through primary prevention with human papillomavirus (HPV) vaccination and secondary prevention with cancer screening (pap and HPV testing). However, the majority of the approximately 11,000 new cases of cervical cancer annually are in patients who were infrequently screened or never screened and who never receive the HPV vaccination.^8^ Similar issues exist for breast, lung, and colon cancers: secondary prevention screening tests exist but there are no recommended primary prevention strategies outside of lifestyle modifications, genetic testing, and tissue removal for colon and breast cancers.^9^

Women at the highest risk of cancer development secondary to social determinants of health are the least likely to be screened. This lessens the impact of primary and secondary cancer prevention programs on reducing cancer mortality.^10^ A decrease in annual primary care visits with subsequent increase in episodic emergency department (ED) usage is a potential contributor to this decline in cancer screening in those who face barriers to adequate health care.^11–13^ Furthermore, rising health care costs, geographic barriers, and lack of primary care access contribute to over-utilization of EDs for routine health needs.^13,14^ In parallel, cancer prevention prevalences are lowest among persons who are uninsured or economically disadvantaged without a usual source of primary health care necessitating utilization of the ED for health concerns.^15^ The ED is therefore a non-traditional yet potentially high yield setting in which to intervene and increase cancer screening among high-risk populations.^16^

The ED is an important yet suboptimal safety net for health care and cancer diagnosis.^17^ The electronic health care record (EHR), digital health, and informatics are proposed approaches to identify patients for primary prevention (vaccination) and secondary prevention (screening tests).^16^ Digital health is broadly defined as the use of technology such as telemedicine, mobile devices, and electronic records to improve patient outcomes and health care delivery. Informatics, on the other hand, is the application of information science to health care.^18^ These approaches include automated documentation in the EHR and point-of-care documentation by existing clinical or research staff.^16^ An overstretched health care system and public health infrastructure presents barriers to widespread implementation of these strategies due to competing priorities at non-traditional touchpoints like the ED and the EHR.

### Objectives

Our primary aim was to compare automated EHR cancer prevention documentation at our institution to a brief, point-of-care electronic survey administered in our ED. Specifically, we sought to evaluate the prevalences of female patients documented as being up to date with HPV vaccination and cervical, breast, and colorectal cancer screening. We hypothesize that the EHR, as it is currently structured, is insufficient to provide accurate screening data on these patients. Ultimately, we also aim to apply lessons learned about informatics tools to utilize non-traditional settings, such as the ED, for identifying and intercepting women at high risk of undetected cancer. This study constitutes a data source agreement EHR quality study of EHR plausibility.^19^

## Materials and Methods

### ED pilot survey study

We completed point of care convenience sampling of women greater than 18 years of age presenting to our local institution’s ED from July 6, 2022 to August 5, 2022. Of 111 patients approached, a total of 101 (91%) individuals accepted and participated. Patients were considered irrespective of ED chief complain but patients who were clinically unstable were not approached. A brief digital survey (administered by a medical student, SKS) obtained data on HPV vaccination status and breast, cervical, and colorectal cancer screening. U.S Preventive Services Task Force (USPSTF) recommendations were utilized to determine the proportion of patients up to date on screening for these cancers and HPV vaccination. Demographic variables of insurance status, body mass index (BMI), zip code, education level, and marital status were obtained *via* extraction from the EHR. Race was categorized based on patients self-identifying as White, Black or African American, Asian or Other. Ethnicity was reported as Hispanic or Latino. The study was deemed as exempt by the Institutional Review Board (IRB 22–008703).

### Electronic health record study

We completed a retrospective cohort study of people designated as female and greater than 18 years of age by our EHR who visited the ED at our institution from July 1, 2022 to July 31, 2022. To ensure a comprehensive capture of screening data, we conducted data extraction from multiple sources within the EHR. Our initial method involved data retrieval using CPT (Current Procedural Terminology) codes and Healthcare Common Procedure Coding System (HCPCS) procedure codes from the Unified Data Platform (UDP), which integrates EHR data from multiple databases across geographically separate sites at our institution. Subsequently, a more comprehensive EHR data extraction was performed incorporating integrated EHR health maintenance modules via Epic Clarity.

Based on their age and the time since their last screening, we determined whether these patients were adherent to the screening guidelines according to the USPSTF recommendations. Patients who did not provide authorization for research during their routine consent for health care were excluded from our analyses. This portion of the study was also approved by our Institutional Review Board.

### Screening adherence definitions

**FIG. 1.**
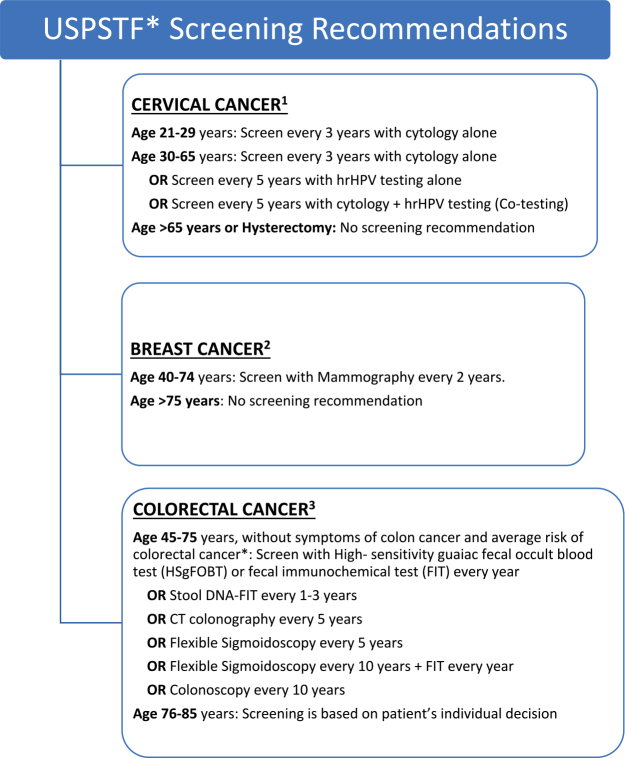
USPSTF* Screening Recommendations. *United States Preventive Services Task Force (USPSTF) is an independent panel of experts in disease prevention and evidence-based medicine. ^1^Cervical cancer screening recommendations were taken from USPSTF 2009 recommendations. ^2^Breast cancer screening recommendations were taken from USPSTF 2023 draft recommendations. ^3^Colorectal cancer screening recommendations were taken from USPSTF 2009 recommendations.

The definitions used to determine screening adherence both on the ED survey and in the EHR is provided in Figure 1 (USPSTF guidelines).

### Analysis

Patients’ demographics, cancer screening adherence, and HPV vaccination rates obtained from the survey were compared with those derived from retrospective data extraction in the EHR. Pearson chi-squared test was used to assess differences in the categorical variables and analysis of variance (ANOVA) *t*-test was used to assess differences between continuous variables across types of data source. Fisher’s exact test was employed to compare the survey data among different age groups. A statistical significance level of 0.001 was used to indicate significance for all comparisons.

The primary outcome of interest was outputs of different digital approaches in evaluating cancer screening and HPV vaccination statuses. The output comparisons were the ED pilot survey, EHR data extraction based on CPT codes, and comprehensive EHR data extraction based on CPT codes and integrated EHR health maintenance modules.

## Results

### Patient demographics

A summary of overall patient demographics is included in Table 1. A total of 101 woman were included in the ED survey data with an average age of 57 years and BMI of 26.7 kg/m^2^. This was similar to the observed average age (59 years) and BMI (28.0 kg/m^2^) in the EHR data. For EHR and survey data, the majority of patients identified as White (80.3% EHR vs. 81.3% in survey), were married (57.0% vs. 53.5%), had Bachelor’s degree education level (27.7% vs. 24.3%) and Medicare insurance status (41.4% vs. 40.6%) for EHR and survey data. The *p* value was <0.001 for insurance status comparisons.

**Table 1.** Overall Patient Demographics at Florida Site

Variable^a^	FL EHR data (*N* = 2934)	ED survey data (*N* = 101)^b^	*p* value
Age (years)	59 (1–105)^c^	57 (21–88)	0.284
BMI (kg/m^2^)	28.0 (10.4–67.9)	26.7 (15.3–46.3)	0.052
Race			0.156
White	2357 (80.3%)	78 (81.3%)	
Black or African American	367 (12.5%)	10 (10.4%)	
Asian	88 (3.0%)	4 (4.2%)	
Other	122 (4.2%)	4 (4.2%)	
Ethnicity (Hispanic or Latino)	180 (6.1%)	7 (7.2%)	0.744
Marital status			0.485
Divorced	262 (8.9%)	14 (13.9%)	
Married	1673 (57.0%)	54 (53.5%)	
Single	678 (23.1%)	22 (21.8%)	
Widowed	307 (10.5%)	10 (9.9%)	
Unknown	14 (0.5%)	1 (1.0%)	
Education level			
Grades 1 through 12, no diploma	302 (18.1%)	14 (20.0%)	0.451
High school graduate	202 (12.1%)	14 (20.0%)	
GED or alternative credential	62 (3.7%)	1 (1.4%)	
Associate degree (*e.g.,* AA, AS)	291 (17.4%)	13 (18.6%)	
Bachelor’s degree (*e.g.,* BA. BS)	465 (27.7%)	17 (24.3%)	
Master’s degree (*e.g.,* MA, MS, MEng, MEd, MSW, MBA)	260 (15.5%)	9 (12.9%)	
Professional degree (*e.g.,* MD, DDS, DVM, LLB, JD)	61 (3.6%)	1 (1.4%)	
Doctorate degree (*e.g.,* PhD, EdD)	33 (2.0%)	1 (1.4%)	
Insurance			<0.001
Private	759 (54.5%)	46 (45.5%)	
Medicaid	10 (0.7%)	3 (3.0%)	
Medicare	577 (41.4%)	41 (40.6%)	
Self-pay	0 (0.0%)	3 (3.0%)	
Other	47 (3.4%)	8 (7.9%)	

^a^ Expressed as mean (range minimum-maximum) or No. (%) of patients.

^b^ For Survey Data, *N* = 101 except for *N* = 96 for Race, *N* = 97 for Ethnicity, *N* = 70 for Education Level.

^c^ Patients younger than 21 years of age in the EHR group would subsequently fall under “not eligible” for screening needs.

### Cancer screening and HPV vaccination status

#### EHR data

Table 2 provides a comparative analysis of overall screening needs for HPV vaccination and cervical breast cancer, and colorectal cancer screening as determined by two different methods of EHR data extraction, and by ED pilot survey.

**Table 2.** **Overall Screening and HPV Vaccination Outputs at Florida Site Utilizing Three Approaches During July 2022** ^a^

Variable^b^	FL EHR data CPT code extraction (*N* = 2934). No. (%) of patients eligible	FL EHR data comprehensive data retrieval^c^ (*N* = 2934). No. (%) of patients eligible	ED survey data (*N* = 101) No. (%) of patients eligible
Cervical cancer screening needed			
Yes	**1447 (93.5%)**	**1202 (77.6%)**	**3 (6.2%)**
No	101 (6.5%)	346 (22.4%)	40 (83.3%)
Missing response	0 (0.0%)	0 (0.0%)	5 (10.4%)
Not Eligible^d^	1386	1386	53
HPV vaccination needed			
Yes	**175 (82.9%)**	**122 (57.8%)**	**3 (33.3%)**
No	36 (17.1%)	89 (42.2%)	5 (55.6%)
Missing response	0 (0.0%)	0 (0.0%)	1 (11.1%)
Not eligible^d^	2723	2723	92
Breast cancer screening needed			
Yes	**1634 (82.2%)**	**1459 (73.4%)**	**9 (14.3%)**
No	353 (17.8%)	528 (26.6%)	47 (74.6%)
Missing response	0 (0.0%)	0 (0.0%)	7 (11.1%)
Not eligible^d^	947	947	38
Colorectal cancer screening needed			
Yes	**1735 (84.8%)**	**1155 (56.5%)**	**19 (22.9%)**
No	311 (15.2%)	891 (43.5%)	59 (71.1%)
Missing response	0 (0.0%)	0 (0.0%)	5 (6.0%)
Not eligible^d^	888	888	18

Bolded data represents screening needs (i.e., screening that was not up-to-date) as identified in each group.

^a^ EHR Data from July 1, 2022 to July 31, 2022; ED Survey data from July 6, 2022 to August 5, 2022.

^b^ *p*-value <0.001 for all comparisons.

^c^ EHR Data Comprehensive Data Retrieval includes data from health maintenance modules from Epic Clarity.

^d^ Not eligible for screening based on age and/or surgical criteria.

Of 2,934 patients seen at the institution’s ED in Florida (from July 1, 2022 to July 31, 2022), 211 were eligible for HPV vaccination, 1,548 were eligible for cervical cancer screening, 1,987 for breast cancer screening, and 2,046 for colorectal cancer screening. Of the 211 patients eligible for HPV vaccination, 122 (57.8%) were deemed to need vaccination according to the comprehensive EHR data (Table 2). Of the 1,548 patients eligible for cervical cancer screening, 1,202 (77.6%) were identified as in need of cervical cancer screening by the comprehensive EHR data. Of the 1,987 eligible, 1,459 (73.4%) were deemed to be in need of breast cancer screening and 1,155 (56.5%) deemed in need of 2,046 patients eligible for colorectal cancer screening. (Table 2).

#### ED survey data

Of 101 patients surveyed in the ED (from July 6, 2022 to August 5, 2022), 48 patients (47.5%) were considered eligible for cervical cancer screening, 9 patients (8.9%) for HPV vaccination, 63 patients (62.4%) for breast cancer screening, and 78 patients (77.2%) for colorectal cancer screening. Three patients (6.2%) were deemed to be in need of cervical cancer screening (Table 2) with 1 of these patients never having received cervical cancer screening. Of the 9 patients eligible for HPV vaccination, 3 individuals (33.3%) were identified as needing the vaccination (Table 2). Among the 63 participants eligible for breast cancer screening, 9 (14.3%) were in need of an updated breast cancer screening (Table 2), with 5 of these patients never having received any form of breast cancer screening. Finally, of 83 eligible women, 19 (22.9%) were in need of an updated colon cancer screening (Table 2) with 9 of these individuals never having received colon cancer screening.

## Discussion

Our study indicates there is a significant discrepancy at our institution between patients’ self-reported screening history and patients’ EHR data. Based on CDC Behavioral Risk Factor Surveillance System estimates of 63.3%, 74.7%, and 84.9% of colorectal, breast cancer, and cervical cancer screening, respectively, of up-to-date outputs in our institution’s geographic regions, the ED pilot survey data administered at the point-of-care performed more closely to what was expected, suggesting that our survey was the more accurate modality.^20^ This considerable gap in the EHR’s ability to accurately identify patients in need of screening demonstrates that point-of-care survey administration is a more believable, plausible, and actionable method in identifying high-risk populations and intervention to improve cancer prevention at our institution at this time. We hypothesize that the gap observed between the two approaches (survey vs. EHR) may be a result of patients receiving care at outside facilities where that data does not consistently crossover into the EHR.

Although there are many opportunities for automated cancer screening documentation from the EHR, the over 50% deemed to be in need of cancer screening based on our EHR data extraction were excessively high to be relied upon when attempting to correctly identify patients who may actually need screening.^19^ Previously, Abar et al. demonstrated the potential of using research associates for point-of-care documentation of cancer screening at five different National Alliance of Research Associates Programs’ sites.^21^ To our knowledge, comparison of a point-of-care documentation survey to an automated EHR documentation approach using CPT codes and health maintenance modules has not been undertaken until this study. There are, however, a small number of studies evaluating cancer screening adherence among ED patients.^16^ Our ED survey study’s patient up-to-date proportions are consistent with patterns observed in the published literature. In one large series involving a variety of ED sites that differ in geographic location and patient volumes, breast and cervical cancer screening compliance obtained from survey administration was 85% and 88%, respectively.^21^ Furthermore, survey results from a study assessing three academic center EDs in Boston, MA showed screening up to date proportions of 88% and 67% for breast and cervical cancer screening, respectively.^22^ Both of these studies, along with our study’s ED pilot survey, show screening up-to-date proportions markedly below the U.S. Federal Government’s *Healthy People 2020* target of 93% for cervical cancer screening.^23^ Our ED pilot survey results are consistent with prior literature and demonstrates the potential use of the ED as a non-traditional setting to help screen patients who may be at higher risk of unidentified malignancy, specifically those without screenings due to inequities in access to routine health care.

The downstream effects of this disparate and fragmented health care among a subset of disadvantaged patients includes delayed cancer diagnosis and potential for advanced stages of cancer at the time of diagnosis.^24,25^ These adverse outcomes further illustrate the need for targeted identification and subsequent intervention of high-risk populations during presentation to the ED as this may be these patients’ only obtainable access to preventative health care. Potential opportunities for such interventions include improvement in cancer screening documentation, improved transition of care practices that utilize patient advocacy mentors, for example, to coordinate the referral processes, and use of on-site research assistants to administer point-of-care surveys to identify those in need of screening. Furthermore, with the introduction of cervical cancer screening through HPV self-collection,^26,27^ real-time ED-based cancer screening is yet another potential modality to intervene in those individuals who lack access to care or maybe overdue on screening needs.

In the outpatient setting, practical difficulties may result if providers rely solely on chart review to determine cancer screening needs. For example, intervention inefficiency can occur when testing does not improve patient outcomes or are not consistent with established guidelines.^28^ Over-referral for screening can lead to overdiagnosis and overtreatment, burdening patients and health care resources.^29^ Furthermore, patient annoyance occurs as a result of repetitive testing, which may arise from a lack of appropriate communication between health care entities or inadequate EHR records. Therefore, this study aimed to explore the opportunities that the ED may provide in detecting individuals in need of cancer screening. Since reliably identifying which patients are in need of cancer screening is a prerequisite to targeting and monitoring intervention impact, this study compared the performance of EHR data with a point-of-care survey. Our results suggest that a point-of-care survey at the time of a patient encounter is a valuable tool in identifying women in need of cancer screening and HPV vaccination.

### Limitations

Regarding EHR data abstraction, we utilized a combination of code-based searches along with expanded methodology to include a more comprehensive EHR data extraction through the health maintenance modules within Epic Clarity. A text-based or language-based search, however, may have identified additional information about screening. The point-of-care survey results are subject to recall bias given the possibility of patients inaccurately recollecting timing of their screening tests and HPV vaccination. Furthermore, the use of a cohort from a quaternary medical center raises concerns about selection bias in the EHR, reducing the generalizability of our study to a broader population while also potentially under-capturing primary health care data. We addressed these limitations through comparison of approaches to best determine believability, plausibility, and next steps for intervention.

## Conclusions

The ED may intercept patients who face barriers to receiving preventative cancer screening and HPV vaccination, especially in light of the EHR’s inability to accurately identify those in need of such testing. Work is underway at our institution to understand the effect on EHR cervical cancer screening output with implementation of point-of-care documentation of cervical cancer screening into the EHR health maintenance module by gynecological providers based on review of outside records and patient’s self-report of cancer screening.

### Clinical relevance statement or summary capsule

Providers must be able to reliably identify patients who are in need of cancer screening. This is a prerequisite to targeting and monitoring the impact of interventions; hence, this study compared the performance of EHR data with a point-of-care survey administered in the ED, which is a non-traditional yet potentially high yield setting for screening patients. Our results suggest that a point-of-care survey at the time of a patient encounter is a more valuable tool in identifying women in need of cancer screening and HPV vaccination than the currently structured EHR at our institution.

## Abbreviations Used

ANOVA: analysis of variance
BMI: body mass index
CPT: Current Procedural Terminology
ED: Emergency Department
EHR: electronic health record
HCPCS: Healthcare Common Procedure Coding System
HPV: human papillomavirus
UDP: Unified Data Platform
USPSTF: United States Preventive Services Task Force

## References

[1] Negoita S, Chen H-S, Sanchez PV, et al. Annual report to the nation on the status of cancer, part 2: Early assessment of the COVID-19 pandemic’s impact on cancer diagnosis. Cancer 2024;130(1):117–127; doi: 10.1002/cncr.35026 37755665 PMC10841454

[2] Cronin KA, Scott S, Firth AU, et al. Annual report to the nation on the status of cancer, part 1: National cancer statistics. Cancer 2022;128(24):4251–4284; doi: 10.1002/cncr.34479 36301149 PMC10092838

[3] Nelson HD, Cantor A, Humphrey L, et al. Screening for Breast Cancer: A Systematic Review to Update the 2009 US Preventive Services Task Force Recommendation. U.S. Preventive Services Task Force Evidence Syntheses, formerly Systematic Evidence Reviews. 2016. 26889531

[4] Melnikow J, Henderson JT, Burda BU, Senger CA, Durbin S, Soulsby MA. Screening for Cervical Cancer With High-Risk Human Papillomavirus Testing: A Systematic Evidence Review for the US Preventive Services Task Force. 2018. U.S. Preventive Services Task Force Evidence Syntheses, formerly Systematic Evidence Reviews. 30256575

[5] Lin JS, Perdue LA, Henrikson NB, Bean SI, Blasi PR. Screening for Colorectal Cancer: An Evidence Update for the US Preventive Services Task Force. U.S. Preventive Services Task Force Evidence Syntheses, formerly Systematic Evidence Reviews. 2021. 34097369

[6] Jonas DE, Reuland DS, Reddy SM, et al. Screening for lung cancer with low-dose computed tomography: Updated evidence report and systematic review for the US preventive services task force. JAMA 2021;325(10):971–987; doi: 10.1001/jama.2021.0377 33687468

[7] Panel PC. Closing Gaps in Cancer Screening: Connecting People, Communities, and Systems to Improve Equity and Access. Updated 02/2022. Available from: https://prescancerpanel.cancer.gov/report/cancerscreening/Part1.html [Last accessed: April 27, 2024].

[8] Prevention NCIDoC. NCI Cervical Cancer ‘Last Mile’ Initiative. Available from: https://prevention.cancer.gov/major-programs/nci-cervical-cancer-last-mile-initiative [Last accessed: April 27, 2024].

[9] Prevention CfDCa. How to Prevent Cancer or Find It Early. Updated 6/20/2023. Available from: https://www.cdc.gov/cancer/dcpc/prevention/index.htm [Last accessed: April 27, 2024].

[10] Prevention CfDCa. Cancer screening Prevalence and Associated Factors Among US Adults. Centers for Diease Control and Prevention. Updated 05/17/2022. Available from: https://www.cdc.gov/pcd/collections/Cancer_Screening_Collection.htm [Last accessed: April 27, 2024].

[11] Ganguli I, Shi Z, Orav EJ, et al. Declining Use of Primary Care Among Commercially Insured Adults in the United States, 2008-2016. Ann Intern Med 2020;172(4):240–247; doi: 10.7326/M19-1834 32016285

[12] Hooker EA, Mallow PJ, Oglesby MM. Characteristics and trends of emergency department visits in the United States (2010–2014). J Emerg Med 2019;56(3):344–351; doi: 10.1016/j.jemermed.2018.12.025 30704822

[13] Coster JE, Turner JK, Bradbury D, et al. Why do people choose emergency and urgent care services? A rapid review utilizing a systematic literature search and narrative synthesis. Acad Emerg Med 2017;24(9):1137–1149; doi: 10.1111/acem.13220 28493626 PMC5599959

[14] Vogel JA, Rising KL, Jones J, et al. Reasons patients choose the emergency department over primary care: A qualitative metasynthesis. J Gen Intern Med 2019;34(11):2610–2619; doi: 10.1007/s11606-019-05128-x 31428988 PMC6848423

[15] Hall IJ, Tangka FKL, Sabatino SA, et al. Patterns and trends in cancer screening in the United States. Prev Chronic Dis 2018;15:E97; doi: 10.5888/pcd15.170465 30048233 PMC6093265

[16] Adler D, Abar B, Wood N, et al. An intervention to increase uptake of cervical cancer screening among emergency department patients: Results of a randomized pilot study. J Emerg Med 2019;57(6):836–843; doi: 10.1016/j.jemermed.2019.07.021 31594738 PMC6904518

[17] Stauder SK, Borkar SR, Glasgow AE, et al. Emergency department visits before cancer diagnosis among women at mayo clinic. Mayo Clin Proc Innov Qual Outcomes 2024;8(3):213–224; doi: 10.1016/j.mayocpiqo.2024.03.002 38596167 PMC11002794

[18] Ronquillo YMA, Korvek SJ. Digital Health. Stat Pearls Publishing. Updated 5/1/2023. Available from: https://www.ncbi.nlm.nih.gov/books/NBK470260/ [Last accessed: April 27, 2024].

[19] Lewis AE, Weiskopf N, Abrams ZB, et al. Electronic health record data quality assessment and tools: A systematic review. J Am Med Inform Assoc 2023;30(10):1730–1740; doi: 10.1093/jamia/ocad120 37390812 PMC10531113

[20] U.S. Department of Health and Human Services CfDCaPaNCI. U.S. U.S. Cancer Statistics Data Visualizations Tool based on 2022 submission data (1999–2020). Cancer Statistics Working Group. Updated November 2023. Available from: https://www.cdc.gov/cancer/dataviz [Last accessed: April 27, 2024].

[21] Abar B, Dalawari P, Ogedegbe C, et al. Identifying cancer screening adherence in the emergency department utilizing research associates. J Emerg Med 2020;59(6):894–899; doi: 10.1016/j.jemermed.2020.07.013 32843249

[22] Ginde AA, Millen JC, Love JS, et al. Compliance with recommended cancer screening among emergency department patients: A multicenter survey. Acad Emerg Med 2008;15(5):483–486; doi: 10.1111/j.1553-2712.2008.00103.x 18439206

[23] Promotion UDoHaHSOoDPaH. Healthy People 2030. 2024. Available from: https://health.gov/healthypeople [Last accessed: April 27, 2024].

[24] Livingood WC SC, Lukens-Bull K, Aldridge P, et al. An elephant in the emergency department: Symptom of disparities in cancer care. Popul Health Manag 2016;19(2):95–101; doi: 10.1089/pop.2015.0118 26760720

[25] McPhail S, Swann R, Johnson SA, et al. ICBP Module 9 Emergency Presentations Working Group. Risk factors and prognostic implications of diagnosis of cancer within 30 days after an emergency hospital admission (emergency presentation): An International Cancer Benchmarking Partnership (ICBP) population-based study. Lancet Oncol 2022;23(5):587–600; doi: 10.1016/s1470-2045(22)00127-9 35397210 PMC9046095

[26] Hawkes D, Keung MHT, Huang Y, et al. Self-collection for cervical screening programs: From research to reality. Cancers (Basel) 2020;12(4); doi: 10.3390/cancers12041053 PMC7226191 32344565

[27] Bohn JA, Fitch KC, Currier JJ, et al. HPV self-collection: What are we waiting for? Exploration of attitudes from frontline health care providers. Int J Gynecol Cancer 2022;32(12):1519–1523; doi: 10.1136/ijgc-2022-003860 36351745

[28] Ooi K. The pitfalls of overtreatment: Why more care is not necessarily beneficial. Asian Bioeth Rev 2020;12(4):399–417; doi: 10.1007/s41649-020-00145-z 33717342 PMC7747436

[29] Adler D, Abar B, Chiao EY. Emergency department-based cancer screening interventions. Emerg Cancer Care 2022;1(1). 10.1186/s44201-022-00012-7 PMC9589631 36312902

